# First person – Rebeca Piñeiro-Sabarís

**DOI:** 10.1242/dmm.052051

**Published:** 2024-09-10

## Abstract

First Person is a series of interviews with the first authors of a selection of papers published in Disease Models & Mechanisms, helping researchers promote themselves alongside their papers. Rebeca Piñeiro-Sabarís is first author on ‘ Deficient GATA6–CXCR7 signaling leads to bicuspid aortic valve’, published in DMM. Rebeca is a postdoc in the lab of José Luis de la Pompa at Centro Nacional de Investigaciones Cardiovasculares, Madrid, Spain, investigating the genetic and molecular mechanisms underlying congenital heart defects.


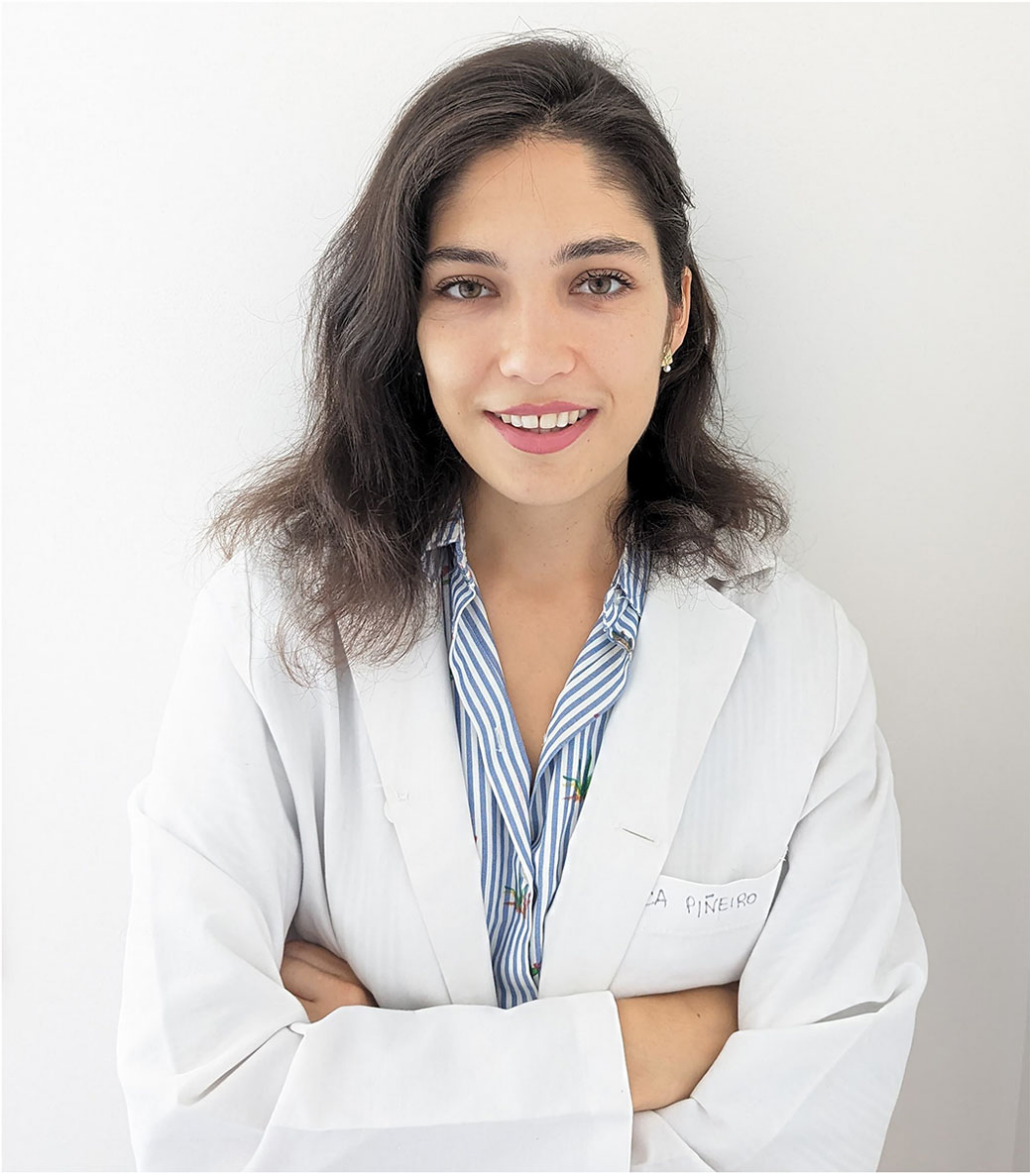


**Rebeca Piñeiro-Sabarís**

**Who or what inspired you to become a scientist?**

Since I was a child, I have harbored a deep interest in understanding the complexity of nature, the etiology of diseases and the remarkable functionality of the human body. My primary motivation was my innate curiosity to understand the human and animal body, and the causes of diseases.

**What is the main question or challenge in disease biology you are addressing in this paper? How did you go about investigating your question or challenge?**

The primary challenge addressed in this paper was the study of the outflow tract (OFT) dimensions. We decided to perform whole-mount immunostaining. Several technical issues were encountered, including tissue fixation, immobilization, clearing and antibody and laser penetration. After resolving these technical challenges, we performed volume rendering and quantified various parameters such as length, diameter and tortuosity. Our findings revealed that the OFT in *Gata6^STOP/+^* mutants is shorter and narrower compared to wild-type OFT.

**How would you explain the main findings of your paper to non-scientific family and friends?**

Bicuspid aortic valve (BAV) is the most common congenital heart defect, affecting between 0.5 and 2% of the population, and is more prevalent in men. BAV consists of two asymmetrical leaflets instead of the usual three symmetrical ones. People with BAV are at higher risk for valve stenosis and calcification, often requiring valve replacement. Previous studies identified *GATA6* as a gene associated with BAV. In our study, we examined the development of the aortic valve and found that *Gata6^STOP/+^* mutants exhibit defects in cell proliferation, migration and invasion, likely underlying the BAV defect. We also discovered that GATA6 is required for the activity of the CXCR7 receptor, which is important for migratory and invasive processes.

Understanding the cellular and molecular mechanisms of GATA6-mediated valve disease could lead to new pharmaceutical therapies, potentially reducing the need for surgical interventions

**What are the potential implications of these results for disease biology and the possible impact on patients?**

These results open new avenues for research into the transcription factor GATA6 and its role regulating the CXCR7 signaling pathway. Understanding the cellular and molecular mechanisms of GATA6-mediated valve disease could lead to new pharmaceutical therapies, potentially reducing the need for surgical interventions. This is particularly significant given the high prevalence of valve disease in the aging population and the associated healthcare costs.

**Figure:**
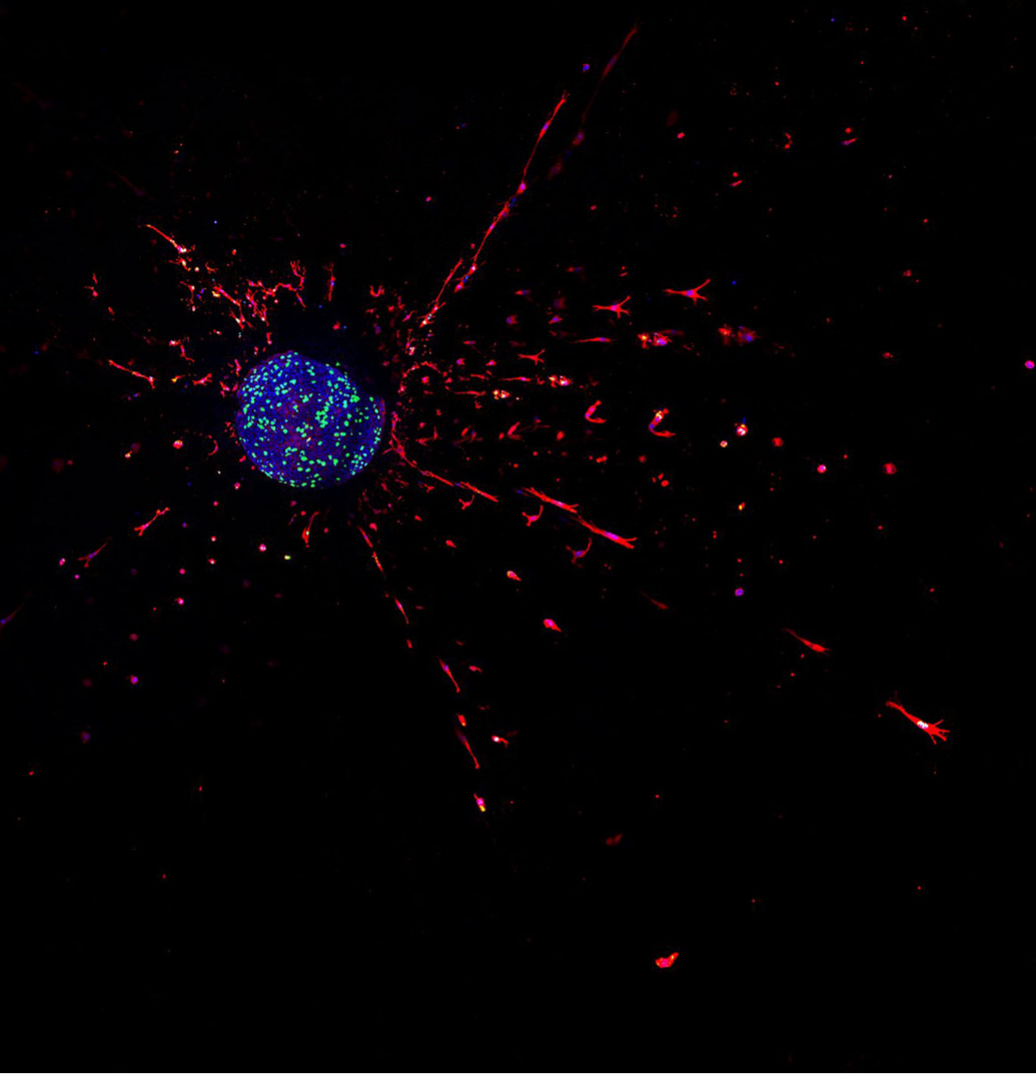
Immunostaining of E11.5 outflow tract tissue cultured for 5 days.

**Why did you choose DMM for your paper?**

We selected Disease Models & Mechanisms (DMM) due to its status as an Open Access biomedical research journal that promotes novel insights into the mechanisms, diagnosis and therapy of human diseases. DMM is committed to publishing rigorously peer-reviewed research with significant translational impact, bridging basic and clinical science. Its interdisciplinary scope and coverage of diverse diseases, along with its leadership by an international team of experts, ensure the publication of high-quality articles.

**Given your current role, what challenges do you face and what changes could improve the professional lives of other scientists in this role?**

I believe that the scientific field is evolving to better support researchers’ personal lives. However, more administrative support staff dedicated to project writing, legal compliance, procurement and general bureaucracy would allow scientists to concentrate more fully on their research projects.

**What's next for you?**

Concerning the project, further investigation is needed to fully elucidate the mechanisms by which the arterial valves develop, which would require several more years.

**Tell us something interesting about yourself that wouldn't be on your CV**

Besides my scientific pursuits, I enjoy playing the double bass in a classical orchestra during my free time.

## References

[DMM052051C1] Piñeiro-Sabarís, R., MacGrogan, D. and de la Pompa, J. L. (2024). Deficient GATA6–CXCR7 signaling leads to bicuspid aortic valve. *Dis. Model. Mech*. 17, dmm050934. 10.1242/dmm.050934 39253784 PMC11413932

